# 4D CT acquisition methods and their anticipated effects on image quality in dynamic CT-scanning of the wrist

**DOI:** 10.1016/j.zemedi.2025.03.001

**Published:** 2025-04-17

**Authors:** Eveline van den Bergh, Iwan Dobbe, Geert J. Streekstra

**Affiliations:** Amsterdam UMC, Amsterdam, The Netherlands

**Keywords:** 4D-CT imaging, Wrist joint

## Abstract

Four dimensional computed tomography (4D CT) has shown to be of value in the diagnosis of motion related wrist joint pathologies. 4D CT can be performed with a variety of acquisition methods. However, the usability of 4D CT is affected by motion and image artifacts associated with the specific acquisition method used. In this literature review we inventorize which acquisition methods exist for 4D-CT imaging at different positions of the human body and what the advantages and disadvantages of each method are. Based on this assessment we formulate recommendations for the 4D CT imaging approach for diagnosing motion related wrist pathologies. We also address future perspectives of image acquisition in 4D CT of the wrist joint. We found that scanning in volumetric mode eliminates irregularity artifacts, while reducing acquisition time per frame reduces blurring artifacts. A reduced acquisition time can be achieved with short gantry rotation times, dual source CT and Reconstruction based on Partial Gantry Rotation (RPGR). However, the effect of RPGR on RPGR-specific artifacts and the effect of different acquisition times on apparent object displacements may be investigated in future research.

## Introduction

Four dimensional computed tomography (4D CT) was introduced to add time as a fourth dimension to images with three spatial dimensions [1]. Since its first introduction 4D CT was adopted by several clinical fields such as imaging of moving thoracic tumors [2] and imaging of moving joints [3]. 4D CT in tumor imaging allows accounting for tumor motion in radiotherapy planning [1] while in joints it can reveal abnormal motion [4]. For some joint pathologies it has shown to facilitate treatment planning to restore normal joint motion [5].

A major research topic in 4D CT of joints is the diagnosis of scapholunate instability or scapholunate ligament tears [6]. 4D CT is valuable in diagnosing such injuries since static radiographs can only show static joint pathology while 4D CT is capable of revealing abnormal motion patterns related to dynamic joint pathology [4]. For the same reason 4D CT is valuable in diagnosing carpal nonunion [5]. A possible alternative to 4D CT for dynamic diagnostics is videofluoroscopy, combined with 3D-2D registration. However, this method suffers from overprojection of the many bones in the wrist which can be expected to limit the precision of motion measurements of wrist bones.

Despite its clear potential, 4D CT suffers from image degradation due to the motion it means to depict [3], [7], [8], [9] similar to a photograph from a camera that will be blurred when the shutter time is too long. A 4D CT image will contain artifacts, with the severity depending on whether the scanning parameters settings are properly adjusted to the motion that is being imaged. Furthermore, because several 4D CT acquisition methods exist, the quality of the images obtained will depend on the method used [10], [11], [12], [13], [14]. Although overviews of 4D CT acquisition methods exist, a detailed review focusing specifically on evaluating wrist motion and the effects on image quality is lacking. The purpose of this review is to give an overview of existing 4D CT acquisition methods, inventorize how its characteristics is expected to affect the image quality in 4D CT of the wrist and to give recommendations for optimal quality 4D CT imaging of the wrist. Since the application of 4D CT in wrist imaging is still upcoming, we decided to expand our search to gain insight from 4D CT imaging methods developed for different positions in the body and for other diagnostic purposes. For example, a large volume of 4D CT research articles was found for monitoring thoracic tumor motion in radiotherapy planning. Like the wrist application, here quantification of the motion of a certain object is a central challenge.

**Figure 1:**
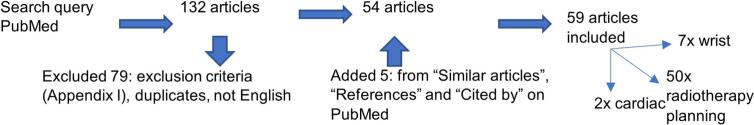
Study selection flow chart.

We performed a literature search using the PubMed database. Fig. 1 gives an overview of the approach. The full search query can be found in Appendix I. The query can be divided into: 1) inclusion criteria for articles that discuss 4D CT; 2) exclusion criteria for articles discussing other imaging techniques such as magnetic resonance imaging and to exclude articles discussing 4D CT applications in which quantification of the motion of an object is not central. Articles discussing cone beam 4D CT were also excluded since it will be difficult to use this modality for the wrist in the standard diagnostic workflow at a radiology department where the cone beam systems used are not designed for diagnostic imaging; and 3) to select 4D CT articles that evaluate or introduce methods to reduce motion artifacts in 4D CT imaging. From the 132 hits, 79 were excluded because they focused on either clinical use or radiotherapy, they focused on combined CT techniques such as positron emission tomography/CT or on segmentation of CT images, or because they did not describe the impact of CT parameters on image artifacts. Other articles were excluded because they were not written in English or because they were duplicates. To find additional references that might have been missed by our search query, we checked the “Similar articles”, “Cited by” and “References” headings on PubMed for the articles that, based on the title, were most specifically focused on comparing multiple acquisition methods. An explanation of these headings can be found in Appendix I. This resulted in a total of 58 included articles (Table 1). From these articles, 7 were dedicated to imaging of the wrist, 49 were dedicated to imaging of tumor motion and 2 to image reconstruction of cardiac scans.

**Table 1:** Overview of used parameter combinations from articles included in this review.

**Mode**	**Gating**	**Motion type**	**Source**	**Reconstruction type**	**Gantry rotation time**	**Article number**
Sequential	Prospective	Periodic	Single	Full or unspecified	<0.5 s	[62]
					0.5 s	[52,56]
					>0.5 s	[51,56]
		Irregular			<0.5 s	[13,35,62,63]
					0.5 s	[27,35,44]
					>0.5 s	[35]
					Unspecified	[12,64]
				Partial	0.5 s	[22]
	Retrospective	Periodic			<0.5 s	[55]*
					0.5 s	[55]*
				Full or unspecified	<0.5 s	[55]*[13,24,62]
					0.5 s	[52], [29], [56], [55], [25], [24], [56], [11], [19], [31], [34], [65], [41]
					>0.5 s	[29], [56], [36], [34], [58], [25], [24], [56], [36]
					Unspecified	[47]**[12], [49], [53]
	None (Cine)	Irregular		Partial	0.5 s	[22]
				Full or unspecified	<0.5 s	[35], [13]
					0.5 s	[35]
					>0.5 s	[35], [7]
		Unspecified			Unspecified	[43]
		Irregular			Unspecified	[39]
Helical	Prospective	Periodic			<0.5 s	[26], [62]
					0.5 s	[52]
		Irregular			<0.5 s	[17], [62]
				Partial	Unspecified	[19]
	Retrospective	Periodic		Partial	<0.5 s	[9]*
					0.5 s	[42]
					>0.5 s	[59]
					<0.5 s	[66]* [10], [11], [63], [27], [20], [17], [18], [67], [54]*
				Full or unspecified	0.5 s	[52], [21], [40]**[29], [50], [27], [14], [16], [28]
					>0.5 s	[29], [34], [33], [15], [28], [46]
					Unspecified	[45]
	None	Periodic			0.5 s	[26]
	Unknown	Unspecified			0.5 s	[21]
					Unspecified	[48]
Volumetric	None	Periodic		Partial	<0.5 s	[8]*[10]
				Full or unspecified	<0.5 s	[8]*
			Dual	Partial	<0.5 s	[23]*
		Irregular	Single		<0.5 s	[57]*[10]
		Irregular			Unspecified	[37]

Overview of CT acquisition methods used in the included articles. Columns from left to right represent 1) the scanning mode used to capture the complete object, 2) the type of gating to synchronize the acquisition with object motion, 3) what type of motion the imaged object performed, 4) the amount of x-ray sources uses to capture the required projection data for each set, 5) whether data from a full or partial gantry rotation was used to reconstruct images, 6) how fast the gantry was rotating, and 7) which articles correspond with the acquisition parameters. * indicates articles dedicated to joint imaging and ** indicates articles dedicated to cardiac imaging, while the unmarked ones are about thorax imaging.

In the next sections, the principles of 4D CT acquisition and reconstruction are explained as well as the different methodological aspects that are of importance for image quality. Subsequently we discuss what the effects are of different acquisition methods on 4D CT image quality and recommend parameter combinations for optimal image quality for 4D-CT wrist imaging.

## 4D CT imaging methods

### Scanning modes

**Figure 2:**
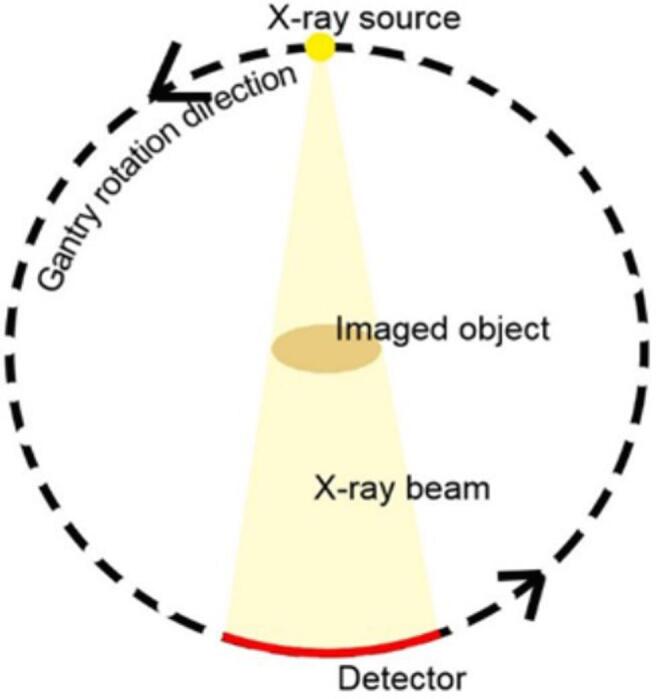
The gantry and its rotation showing the x-ray source and detector with the x-ray beam passing through the imaged object.

In a CT-scanner the x-ray source-detector combination is part of the gantry (Fig. 2). The patient is placed on a CT couch, which can move along the gantry’s z-axis through the CT scanner bore. This movement of the couch is required when the detectors’ z-coverage is smaller than the size of the anatomy of interest in the z-direction. Couch movement is what distinguishes the following three scanning modes:

#### Sequential mode

**Figure 3:**
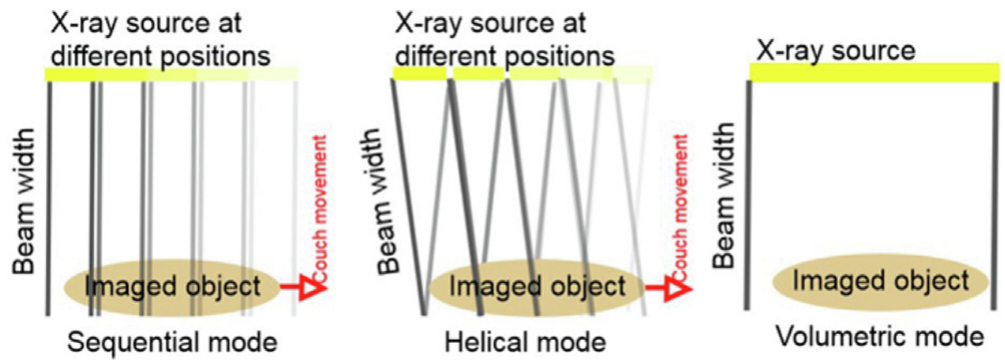
Three different scanning modes with movement of the imaged object on the CT couch relative to the x-ray .

source. Left: Sequential mode with stepwise couch movement. Middle: Helical mode with continuous motion of the bench and helical motion of the X-ray source around the imaged object. Right: Volumetric mode where the longitudinal size of the scanner's field of view is larger than the longitudinal size of the scanned object and no bench movement is required

In sequential mode, the CT couch with the imaged object translates in sequential steps through the CT scanner (Fig. 3). During each sequential step, part of the anatomy of interest is scanned at different stages of the object’s motion cycle. This is repeated until enough raw data is collected for reconstruction of 3D CT scans at multiple motion phases that altogether form the 4D CT scan [15].

#### Helical mode

The helical mode is also called spiral mode [16]. In this mode, the CT couch with the object to be imaged move at a constant speed through the CT scanner, thereby scanning the object volume in a helical fashion (Fig. 3). Couch movement is typically slow, although fast methods have also been reported [17], [18], [19]. In the slow method, the couch moves slow enough to acquire sets of projection data at different phases of the motion cycle while the object travels through the scan volume [15], [20], [21], [22]. In this way, a full image of the object at each motion phase within a cycle can be reconstructed. The fast method uses repeated scans to acquire projection data in sufficient phases and positions [17], [18], [19].

#### Volumetric mode

In this mode, also called çine mode (Mutaf et al [25]), the CT couch is in a fixed position on which multiple 3D scans are made (Fig. 3). This mode requires a CT scanner with a relatively wide detector, so that the z coverage is large enough to capture a 3D image of the entire object of interest in one rotation [8], [10], [23]. The collection of 3D scans acquired at different time points together form a 4D CT scan. Scanners with a z-coverage of up to 16 cm exist [8]. In both sequential and helical mode, the scanning protocol needs to be correlated to a cyclic object motion to allow for retrospective sorting of acquired data in the reconstruction phase. [9], [24], [25]. This explains the name “respiratory correlated CT”, given to the technique by the authors who first introduced it in 2003 [1]. The correlation is done by either retrospectively or prospectively monitoring the object’s motion and triggering the CT acquisition accordingly. This will be explained in detail in the next section “Gating”. There is not unequivocal evidence that helical scanning results in CT images with the least artifacts. There was one research group which found helical mode to perform better, but in those scans the temporal resolution of the helical scan was higher than in the scans in sequential mode [11]. Since in volumetric mode one gantry rotation provides a complete 3D image of the object, the volumetric mode does not require the object to move periodically to capture a complete motion.

### Gating

If the detector is not wide enough for the object to be imaged entirely at once, the motion of the object needs to be tracked, to determine when image acquisition should take place (prospective gating), or to select which part of the projection data should be used for image reconstruction of an image in a certain motion phase (retrospective gating).

#### Prospective gating

This gating type is typically done in sequential mode. The motion is tracked, and CT acquisition is triggered when regular motion is suspected, which opens the gate for image acquisition. It thereby tries to predict when a regular motion pattern is upcoming and tries to skip imaging irregular motion patterns. When enough data is acquired in one couch position, the couch moves to the next position to acquire the next set of 2D slices.

#### Retrospective gating

This type of gating is done both for sequential and helical mode [18]. Image acquisition is continuous, while the motion cycle is monitored. Each projection dataset is labelled with the spatial position of the couch and the phase of the motion cycle [18], [26], [27]. Thus, open gates are retrospectively appointed to different parts of the projection data, to reconstruct a complete 4D image. In thorax scanning, monitoring was usually done by system where a camera monitors the position of a marker that moves along with the object [28], [29], [30]. Other methods also exist such as using a belt around the (phantom) torso to track breathing motion [28], [30] or spirometry [31]. Gating in cardiac 4D CT scanning is done based on the electrocardiogram [32]. Several researchers have shown that when gating is used, CT images will contain less artifacts than when no gating is used [13], [33], [34]. Additionally, the strengths of prospective gating compared to retrospective gating are a reduced dose delivery (especially if not all motion phases are required) and reduced image artifacts due to the selection of regular motions, at the cost of scanning time [12], [13], [22], [26], [35]. Authors of one article found that the limited data with prospective gating might reduce the image quality compared to retrospective gating. However, in their study, the imaged motion was forced to be periodic. Thus, the necessity to skip imaging irregular motion patterns did not apply [26].

### Image sorting

**Figure 4:**
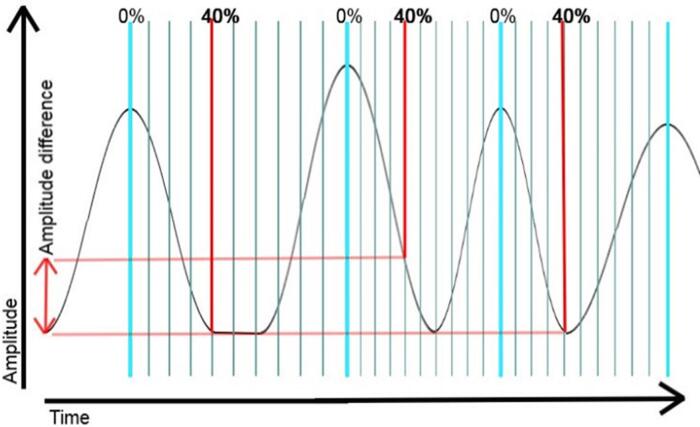
Artefacts arise in phase based sorting when motion is irregular. In this example, 40% of the second cycle does not match the amplitude at 40% in the first and third cycle.

One 3D image is generally built up of several projection data sets, acquired at different couch positions. Projection data sets of one specific phase in the motion cycle are acquired at different couch positions, whether that is done sequentially or helically. For different phases of the motion cycle, these data sets are sorted in image bins [33], [36], [37]. For example, the motion cycle is divided into 10 separate phases (0–10%, 10–20% etc. of one complete motion cycle) and the datasets acquired at different couch positions are sorted accordingly, based on the motion tracking data described above. Thus, one bin collects projection data to reconstruct a series 2D slices that build up a 3D image and the 3D images from all bins together form the 4D image. The standard sorting method is based on the phase of the motion cycle [18], where projection datasets are sorted according to the amount of time that has passed after the start of a certain motion cycle (Fig. 4). As an alternative an amplitude or displacement sorting method can be used [18], where projection datasets are sorted according to the amplitude of the motion tracking device. Other sorting methods also exist such as velocity or volume sorting, or combinations of various sorting types [16], [37], [38], [39], [40], [41].

Although data is available on the comparison of different sorting methods [14], [42], [43], [36], [37], [38], [39], no decisive answer can be formulated about which sorting method results in the least image artifacts in irregular motion. This is because different authors compared different selections of methods or came to different conclusions with different comparison methods. For a more extensive description on image reconstruction methods please refer to [41], [44], [45] for techniques on selecting projection images for image reconstruction, and to [39], [43], [46], [47], [48], [49], [50] for approaches to also reduce motion artifacts.

### Object motion characteristics influencing image quality

Image quality is affected by the amount of motion of the object during acquisition [24], [26], [51], [52], [53]. This is true for all scanning modes. Artifacts that occur due to motion of the imaged object are called motion artifacts [8], [9]. Motion artifacts manifest themselves as blurring or ghosting but can also be present with apparent movement or size changes of the imaged object [8], [11]. Additionally, the sequential and helical modes require the motion to be close to periodic to minimize irregularity artifacts, characteristic for those modes. However, the direction of the motion [8], [54], [55] and the object’s size [56] influence artifact severity as well.

Sonier *et al.* (2020) showed that artifact severity increases when the imaged object’s motion amplitude exceeds its diameter [53]. Dobbe *et al.* (2019) showed that artifacts are increased when the object rotates opposite to the direction of the scanner’s gantry, as compared to when it rotates in the same direction [8]. Choi *et al.* (2013) showed that when using volumetric mode, artifacts are reduced when rotation of the object is around the scanner’s y-axis (vertical axis), compared to rotation around its z-axis (axis following the CT couch) [57].

### Artifacts characteristic for sequential and helical mode

**Figure 5:**
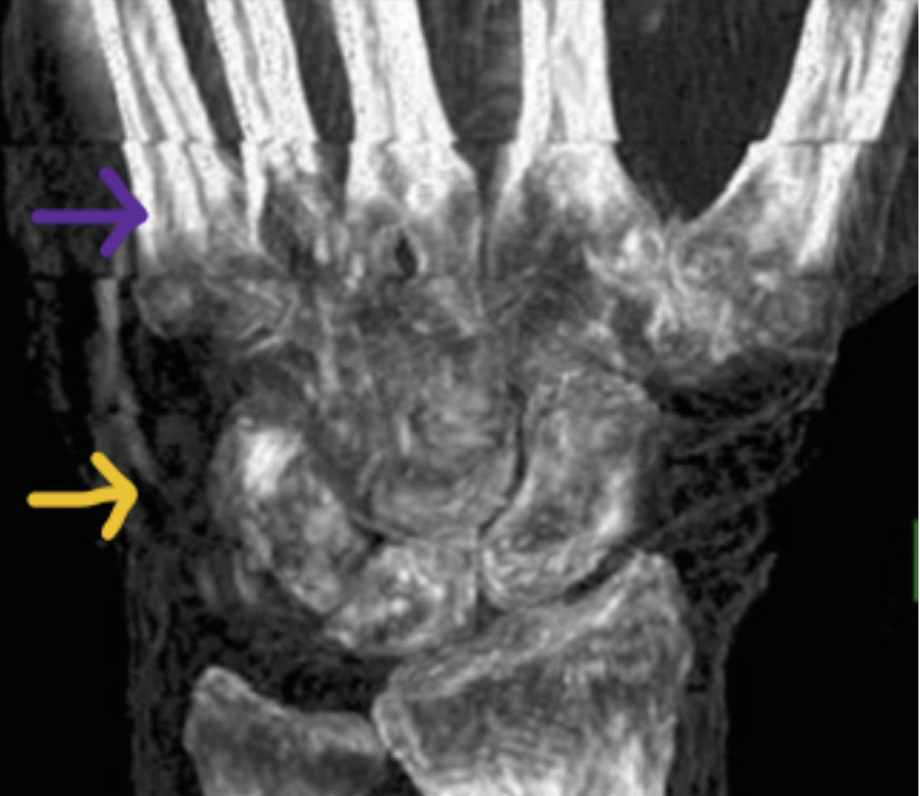
Banding artefacts in wrist with more severe artefacts in the metacarpal region (purple) than in the carpal region (yellow) (Tay et al. 2007).

Several image artifact types can be distinguished in 4D CT. Banding artifacts only occur in sequential mode and helical mode, but not in volumetric mode [16], [21], [23], [37], and can be considered a type of irregularity artifact. They occur when projection datasets within a bin are not acquired at the exact same phase in the motion cycle as shown in Fig. 4, which causes malalignments of consecutive image slices (z), hence banding artifacts in xz and yz planes, as is visible in Fig. 5. [9], [18]. Banding artifacts do not occur in volumetric mode, since here a full 3D image can be captured within one gantry rotation.

### Artifact reduction

As mentioned above, banding artifacts can be avoided by scanning in volumetric mode as long as the anatomy to be imaged fits within the Field View of the scanner. For sequential and helical mode, banding artifacts can be reduced when the imaged object motion is closer to periodic. Another important measure to minimize artifacts in the spiral mode is to choose a pitch low enough to illuminate every voxel of the patient's anatomy during an entire motion cycle [21]. However, a given scan length requires a minimum pitch value at a given rotation time and detector width. If the desired scan length cannot be achieved with the required low pitch value, the motion frequency and pitch should be increased to ensure both illumination of every voxel and achievement of the scan length. For all three scanning modes the acquisition time is of profound influence [24], [26], [51]: smaller acquisition times reduce motion artifacts.

**Figure 6:**
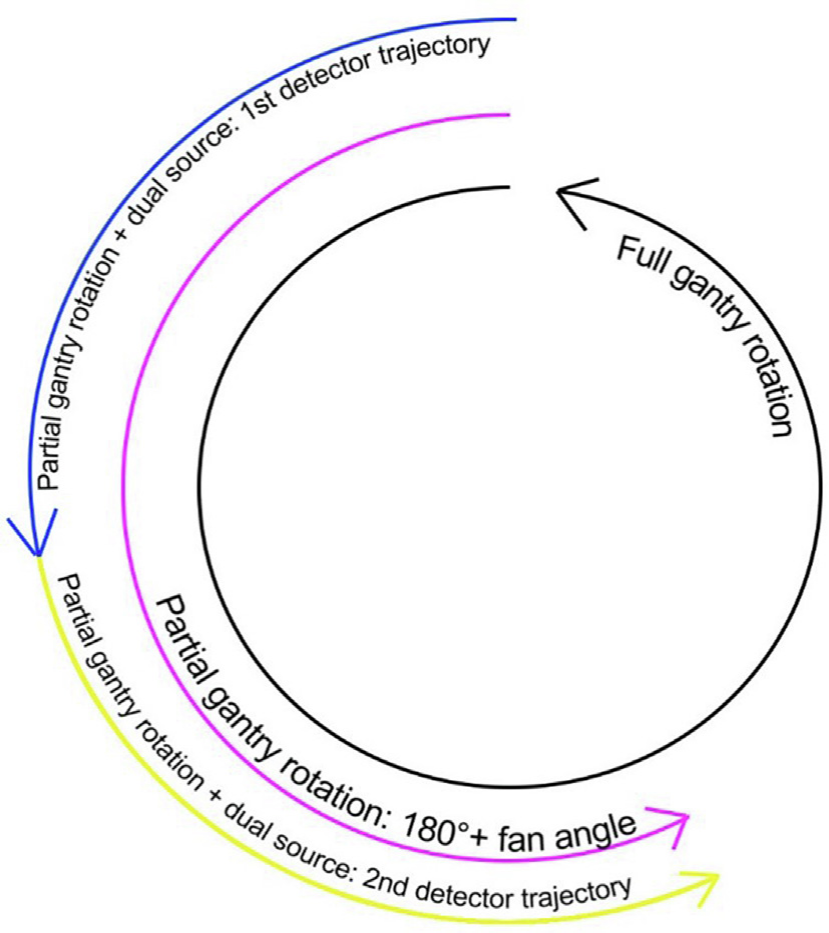
Possible trajectories of CT X-ray tube rotation in 4D-CT imaging. The inner black circle representing a regular full gantry rotation. The purple represents the trajectory when only part of the revolution is used for image reconstruction and the blue and yellow show the trajectories of both tubes in dual source CT.

The acquisition time is dependent on a few parameters. Primarily, it is determined by the gantry rotation time [7], [18], [55], [56], [58]: the time it takes for the gantry to complete one rotation around the z-axis. The gantry revolution time was in the range [0.25, 1.5] s in the reviewed articles. Next, the acquisition time can be reduced by only using projection data of part of a gantry revolution for image reconstruction. These images are reconstructed based on a partial gantry rotation (RPGR). Usually, projection data of 180° + fan angle is used, where the effective fan angle is dependent on the size of the imaged object [8], [22], [59]. Finally, the acquisition time can be reduced by using multiple x-ray sources, for example with dual source CT. Then, each source is responsible for part of the used projection data for image reconstruction, so that a smaller gantry rotation angle can be used to acquire and reconstruct images [23], [32] (Fig. 6).

## Discussion

In the former sections we presented an overview of 4D CT acquisition methods and focused on the severity of image artifacts and related acquisition parameters for each of these methods. Although the use of 4D CT in wrist diagnostics is upcoming, only a small amount (7/58) of the included articles was dedicated to the wrist. Other fields of 4D CT research were included to be able to investigate the use of alternative approaches for possible motion imaging of the wrist.

A major part of the included articles focused on breath-related tumor motion for radiotherapy planning (49/58). This application is comparable to the application of 4D CT in the wrist in the sense that they are both centered around motion quantification of a certain object. However, a difference between the two applications is given by the fact that thoracic imaging focuses on much larger objects compared to wrist imaging. The smaller size of the wrist makes it possible to acquire images of the wrist in volumetric mode, as a smaller scanner z-coverage is sufficient to fit the whole object.

There are no discrepancies among reviewed articles concerning the way to improve image quality in 4D CT imaging: acquisition times need to be short and the volume that can be imaged in one gantry rotation should be large enough to capture the full object. As Yaegashi *et al.* state, motion blur depends on the amount of motion that takes place during acquisition of one frame [51], and volumetric mode eliminates banding artifacts and does not require periodic motion of the scanned object [23]. Low motion velocity could also increase image quality, also when moving in a periodic fashion. Whereas in 4D CT of the ‘wrist’ motion is sometimes imposed by a motion imposing device [23], in practice it is difficult for patients to perform a periodic [23] movement at a low constant velocity [57]. Additionally, not every motion is under voluntarily control, especially in patients, due to pain or high-velocity clicks of the wrist bones [23], [61].

**Figure 7:**
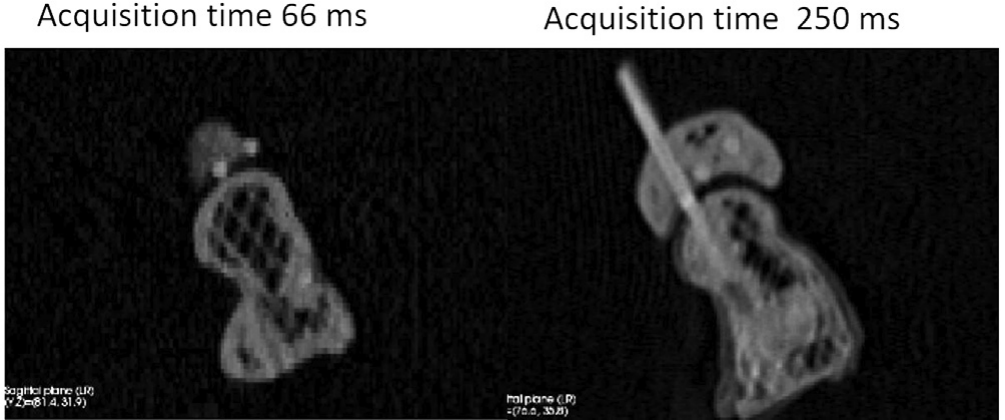
A comparison between a wrist phantom rotating at 0.1 rps imaged with a temporal resolution of 66 ms (left) and 250 ms (right). Motion artifacts are clearly visible on the right side.

Improvement of the 4D CT technique should therefore be sought in improving the hardware and required software. Faster acquisition speeds can be reached with shorter gantry rotation times [7], [18], [25], [58]***,*** by using Reconstruction based on a Partial Gantry Rotation (RPGR) images [23], [55] and by using multiple x-ray sources [23], [54]. A dual source scanner with full gantry rotation time of 0.28 s using partial gantry revolution for reconstruction could reach a temporal resolution of down to 75 ms [23]. Fig. 7 shows the difference between image quality when a wrist phantom rotates at 0.1 rps and is imaged with a temporal resolution of 66 ms and 250 ms. These images are taken from unpublished work from our research group. The carpal bones have been imaged volumetrically by a few research groups. Choi *et al.* were able to do so with a dual source scanner with a longitudinal field of view (LFOV) of 3.8 cm [57]. However, they were unable to use the dual source functionality, since the available dual source protocols required CT couch movement, which is incompatible with volumetric 4D CT imaging. In 2011, Leng *et al.* managed to use the dual source functionality on the same type of CT scanner to image a cadaveric wrist in volumetric mode [23]. They were able to reach a temporal resolution of 75 ms by applying the combination of short gantry rotation times, dual source CT and RPGR. In 2019, Dobbe *et al.* used a CT scanner with a relatively wide LFOV of 5.8 cm to make volumetric 4D CT acquisitions of carpal bones in a phantom wrist [8]. This makes it easier to position the target structures in the scanner’s FOV, even during motion of the target structures [8]. The CT scanner used by Dobbe *et al.* (2019) could also be used with dual sources, which enables a temporal resolution of 66 ms, once a dedicated protocol is available. This allows imaging the carpal bones in the wrist volumetrically and with high temporal resolution, which in turn allows for imaging of wrist motion at a more physiological speed. The physiological speed of carpal bones may differ between different tasks. Whether using a non-physiological speed hampers detecting clinically relevant abnormalities is an interesting subject for future study.

Reducing acquisition time may have some drawbacks when RPGR images are used. Dobbe *et al.* found gantry trajectory dependent deformation of images when using RPGR. They suspect this was due to mechanical imperfections of the gantry mechanics [8]. Teixeira *et al.* showed that even though using RPGR images reduces motion artifacts in scanning a rotating disk phantom, it also increases image noise by 23% compared to reconstruction based on full gantry rotation [55]. However, they used lower radiation doses than what other authors safely used in wrist scanning [23], [55], which also influences the noise. One could question if this noise increase is really problematic in the wrist, since Leng *et al.* were able to produce images of a wrist with high diagnostic confidence even with reduced tube current [23], [60]. They did not explain how that was possible, but it might be due to the small volume of the wrist and the corresponding low attenuation of x-ray radiation. Additionally, Dobbe *et al.* state that in their quantitative analysis of 4D CT data, the errors caused by noise were small in comparison with errors caused by motion artifacts, which indicates that noise increase has limited impact. Should noise be problematic, its effect could be reduced by increasing the tube current to keep the delivered photon flux per acquisition constant, as Mutaf *et al.* had to do in their research dedicated to monitoring tumor motion [25]. A problem with increasing the tube current is the increased radiation dose. For CT scans of the wrist this may be less of a problem compared to thorax imaging, as the wrist and its surroundings are less radiosensitive [23]. Leng *et al.* were able to produce high quality images of the wrist with effective skin doses ten and sixty times lower than the threshold at which the skin starts to respond [23]. In practice, however, we expect that a tenfold higher dose will never be reached . Cancer risks were also relatively low due to the low radiation weighting factor of the tissues in the wrist and thus there should be some room to adjust tube current to improve image quality if necessary. Leng *et al.* report that they were able to produce 4D CT images of a moving wrist without motion artifacts. However, they scored images based on artifacts visible to the naked eye [23]. Dobbe *et al.* showed that motion artifacts like blur can show themselves in the form of image deformation resulting in apparent motion of objects relative to one another [8]. For example, they found a decrease in apparent motion of the capitate relative to the lunate from 12.63° at a rotation rate of 0.4 rps to 3.17° at 0.15 rps. Dobbe *et al.* did use a lower temporal resolution in their 4D CT scans than Leng *et al.* did and it is therefore interesting to study image deformation due to motion artifacts with higher temporal resolutions.

Also, the use of RPGR requires future research. While it can reduce the acquisition time by up to about a factor two in wrist imaging, it was also found to introduce new types of artifacts. The deformation artifacts found by Dobbe *et al.* [8] seem the most concerning, as the increased noise found by Teixeira *et al.* [55] might be suppressed by increasing tube current. Even though the use of RPGR images is standard in cardiac imaging, we found no cardiac 4D CT research that studied the effects of the use of it. It is valuable to learn why and when partial scan artifacts arise, if it has indeed a mechanical cause and if this is the case with all scanners or only a certain type of CT scanners. This study focuses specifically on acquisition methods to improve image quality in volumetric 4D CT. While this study shows that this is a promising step in minimizing motion artifacts, other strategies are also available. Advanced algorithms or deep learning based approaches, for instance, can play a complementary role in suppressing artifacts or accurately imaging motion of the wrist. Developments in image reconstruction techniques, such as improved algorithms for image sorting [40], [43], [45] and automated algorithm approaches for image interpretation [68], [69], have demonstrated significant potential in this regard. Combining optimized acquisition protocols with such post-processing strategies could further enhance both image quality and clinical applicability.

## Conclusions

The reviewed literature describes three modes in which 4D CT can be performed: sequential mode, helical mode and volumetric mode. In all three approaches motion artefacts occur. However, the use of a volumetric mode eliminates the presence of banding artefacts found in sequential and helical mode. Therefore, the preferred mode in wrist 4D CT is volumetric mode. Additionally, the motion artifacts can be reduced by reducing the acquisition time per frame. This can be achieved with short gantry rotation times, dual source CT and RPGR. However, the effect of RPGR on RPGR-specific artifacts and the effect of different acquisition times on apparent object displacements should be investigated. Finally, a larger z-coverage can attribute to easy positioning of the imaged object in the scanner’s field of view.

## CRediT authorship contribution statement

**Eveline van den Bergh:** Writing – original draft, Project administration, Methodology, Formal analysis, Conceptualization. **Iwan Dobbe:** Writing – review & editing, Supervision, Software, Methodology, Formal analysis, Conceptualization. **Geert Streekstra:** Writing – review & editing, Supervision, Methodology, Conceptualization.

## Declaration of competing interest

The authors declare that they have no known competing financial interests or personal relationships that could have appeared to influence the work reported in this paper.

## Appendix I

***A. Including Main Topic 4D CT***

((“four dimensional computed tomography”[mesh] OR “4D CT”[tiab] OR “four dimensional CT”[tiab] OR “four dimensional computed tomography”[tiab]) OR (“four dimensional”[tiab] AND “computed tomography”[tiab]) OR (“four dimensional”[tiab] AND “CT”[tiab])).

***B. Exclusion Part***

NOT (“c-arm”[tiab] OR “Compton scatter”[tiab] OR “angiography”[tiab] OR “pet”[tiab] OR “spect”[tiab] OR “mri”[tiab] OR “skull”[tiab] OR “intracranial”[tiab] OR “tomosynthesis”[tiab] OR “dosimetr*”[ti] OR “cone beam computed tomography”[mesh] OR “cone beam”[tiab] OR “Magnetic Resonance Imaging”[mesh] OR “Positron Emission Tomography Computed Tomography”[mesh] OR “Positron-Emission Tomography”[mesh] OR “Tomography, Emission-Computed, Single-Photon”[mesh] OR “dual energy ct”[tiab] OR “X-Ray Microtomography”[mesh] OR “micro CT”[tiab] OR “perfusion”[tiab]).

***C. Inclusion Part***

AND (“motion artifact”[tiab] OR “motion artifacts”[tiab] OR “motion artifacts”[tiab] OR “banding artifact”[tiab] OR “banding artifacts”[tiab] OR “dual source”[tiab] OR “gantry rotation”[tiab] OR “scan parameters”[tiab] OR “temporal resolution”[tiab] OR “helical 4D CT”[tiab] OR “spiral 4D CT”[tiab] OR “cardiac gated”[tiab] OR “ecg gated”[tiab] OR “motion imaging”[tiab] OR “Prospective gating”[tiab] OR “retrospective gating”[tiab]).

***D. PubMed headings used for additional articles***

Similar articles  A functionality by PubMed that shows articles related to the viewed article.

Cited by  A functionality by PubMed that shows articles that have cited the viewed article.

References  A functionality by PubMed that shows the articles that were cited by the viewed article.
